# Mitochondrial Genome-Encoded Long Noncoding RNA *Cytochrome B* (Lnc*CytB*) and Mitochondrial Ribonucleases in Diabetic Retinopathy

**DOI:** 10.3390/biomedicines12081637

**Published:** 2024-07-23

**Authors:** Jay Kumar, Pooja Malaviya, Renu A. Kowluru

**Affiliations:** Ophthalmology, Visual and Anatomical Sciences, Wayne State University, 4717 St. Antoine, Detroit, MI 48201, USA

**Keywords:** diabetic retinopathy, long noncoding RNA, Lnc*CytB*, mitochondria, mitochondrial ribonuclease P

## Abstract

Aim: Hyperglycemia damages mitochondria and downregulates transcription of mtDNA-encoded genes and the long noncoding RNA Lnc*CytB*, causing mitochondrial genomic instability. The genes encoded by mtDNA are transcribed as large polycistronic transcripts, and the 5′ ends of precursor tRNAs are processed by mitochondrial-targeted ribonuclease P (MRPPs). Our aim was to investigate the role of MRPP1 in the downregulation of Lnc*CytB* in diabetic retinopathy. Methods: Using human retinal endothelial cells incubated in 20 mM D-glucose for 96 h, the gene expression and mitochondrial localization (immunofluorescence) of MRPP1 and the interaction between MRPP1 and Lnc*CytB* (determined by RNA-FISH and RNA immunoprecipitation) were quantified. The results were confirmed in retinal microvessels from streptozotocin-induced diabetic mice and from human donors with documented diabetic retinopathy. Results: Compared to normal glucose, high glucose decreased mRNA and mitochondrial localization of MRPP1 and its interaction with Lnc*CytB*. While MRPP1 overexpression prevented glucose-induced decrease in MRPP1–Lnc*CytB* interaction, Lnc*CytB* expression and mitochondrial damage (reduction in protective nucleoids in mtDNA), MRPP1-siRNA further worsened them. Similar results were obtained from retinal microvessels from diabetic mice and from human donors with diabetic retinopathy. Conclusions: Downregulation of MRPP1 in diabetes suppresses Lnc*CytB* transcription, resulting in mitochondrial functional and genomic instability, ultimately leading to the development of diabetic retinopathy. Thus, preventing MRPP1 downregulation has the potential to inhibit retinopathy and prevent the fear of vision loss in diabetic patients.

## 1. Introduction

Diabetes is now considered an epidemic of the twentieth century and is projected to impact over 1.3 billion people globally in the next 25–30 years [1,2]. Hyperglycemia alters many biochemical and metabolic pathways, and these metabolic abnormalities lead to diabetic complications; retinopathy is one of the major microvascular complications of diabetes [3,4]. Although the pathogenesis of this blinding disease is still not clear, mitochondrial dysfunction is considered to play a central role; damaged mitochondria accelerate capillary cell apoptosis [3,5]—a phenomenon which precedes the development of diabetic retinopathy [6].

Mitochondria are double-membrane organelles with a porous outer membrane and an inner membrane containing essential components of the electron transport chain, which is involved in ATP synthesis [7,8]. Protons are pumped into the inter-membrane space by the transfer of electrons through complexes I, III and IV, and this generates a proton gradient to produce ATP. However, some electrons leak out and interact with molecular oxygen to form superoxide radicals [9,10]. During oxidative phosphorylation, complex I and complex III of the electron transport chain system are the main sites for the generation of reactive oxygen species (ROS) [11,12], and in diabetic retinopathy complex III activity is inhibited [13]. Mitochondria are unique cellular organelles with their own DNA, and compared to nuclear DNA, mitochondrial DNA (mtDNA) is very small, consisting of only ~16,500 base pairs [14,15]. Although mitochondrial homeostasis involves over 1500 proteins, genes for only 13 proteins are encoded by mtDNA. These mtDNA-encoded proteins are solely involved in the functioning of the electron transport chain, and importation of nuclear DNA-encoded proteins is critical for the maintenance of mtDNA through replication and repair [16,17].

Recent technological advancements have demonstrated that noncoding RNAs can also regulate gene expression, and among several types of noncoding RNAs, long noncoding RNAs (lncRNAs) are endogenous RNAs with transcript lengths of more than 200 nucleotides which do not have an open reading frame for coding proteins [18,19]. Aberrant expression of lncRNAs is implicated in several diseases, including cancer and Alzheimer’s disease, and it is also associated with many metabolic abnormalities observed in diabetic retinopathy [20,21,22,23]. Out of over 30,000 lncRNAs identified thus far, only three major lncRNAs are encoded by mtDNA, namely, *ND5*, *ND6* and *Cytochrome B* (Lnc*CytB*), corresponding to the regions complementary to the mitochondrial *ND5*, *ND6* and *Cytochrome B* genes, respectively [24,25]. We have recently shown that although hyperglycemia has no significant effect on Lnc*ND5* and Lnc*ND6*, Lnc*CytB* expression is significantly decreased in the retina and its microvasculature in diabetes, contributing to mtDNA instability in diabetic retinopathy [26].

Mitochondrial DNA is a double-stranded circular DNA molecule containing one heavy and one light strand, and the genes are transcribed as large polycistronic transcripts covering almost the entire length of each strand [27,28]. These precursor transcripts undergo processing to form functional RNAs, and the first step in processing mitochondrial precursor tRNA is the cleavage of the 5′ leader, which is catalyzed by mitochondrial ribonuclease P proteins (MRPPs) [27,29]. These ribonucleases lack an RNA component essential for catalytic function and initiate the maturation of the precursors by cleaving at the 5′ ends of the tRNAs [27,30]. MRPPs are also implicated in the processing of mitochondrial genome-encoded lncRNAs. In humans, MRPPs are composed of three subunits, and, among these, MRPP1 is considered necessary for the accumulation of mtDNA-encoded lncRNAs and for the maturation and abundance of Lnc*CytB*, Lnc*ND5* and Lnc*ND6* [24,27]; however, the role of MRPP1 in the regulation of Lnc*CytB* expression is unclear.

The goal of this study was to investigate the role of MRPP1 in Lnc*CytB* expression in diabetic retinopathy. Using human retinal endothelial cells (HRECs), interactions between MRPP1 and Lnc*CytB* and the effect of regulation of *MRPP1* on Lnc*CytB* expression and mitochondrial stability were determined. The results were confirmed using retinal microvessels from streptozotocin-induced diabetic mice and from human donors with documented diabetic retinopathy.

## 2. Methods

Retinal endothelial cells: Human retinal endothelial cells from the 5th–8th passage were incubated in 5 mM D-glucose (NG) or 20 mM D-glucose (HG) for 96 h, and as an osmotic/metabolic control, HRECs in 20 mM L-glucose (L-Gl), instead of 20 mM D-glucose, were included in each experiment [26,31]. A group of cells from the 5th–6th passage were transfected with an *MRPP1*-overexpressing pcDNA3.1 plasmid (cat. no. OHu09980D; GenScript Biotech, Piscataway, NJ, USA) or with *MRPP1*-siRNA (cat. no. s29782; Invitrogen, Waltham, MA, USA) or with plasmids of the gene encoding for the mitochondrial superoxide scavenging enzyme manganese superoxide dismutase, *Sod2* (cat. no. SC127816; OriGene Technologies, Rockville, MD, USA) using TurboFectin 8.0 transfection reagent (cat. no. TF81001; OriGene Technologies). Cells transfected with an empty vector or with scrambled control RNA were used as controls. Each experiment was performed with cells from the same batch/passage and was repeated in 3–4 different cell preparations. *MRPP1* and *Sod2* overexpression were confirmed by quantifying their respective gene transcripts.

Mice: Mice (~20 g BW, male) overexpressing the *Sod2* gene or wild type (WT) were made diabetic by intraperitoneal injection of streptozotocin (55 mg/kg body weight, solubilized in 50 mM citrate buffer (pH 4.5)) for four consecutive days. Three days after the last injection, mice with blood glucose levels >250 mg/dL were considered diabetic and were maintained diabetic for six months. Age-matched nondiabetic *Sod2* and WT mice were used as their respective controls [13]. The treatment of the animals conformed to the Animal Research: Reporting of In Vivo Experiments (ARRIVE) guidelines, and the protocol was approved by Wayne State University’s Animal Care and Use Committee (protocol no. AIC 21-11-4186).

For retinal cryosections, freshly harvested eyes were fixed in 2% paraformaldehyde in phosphate-buffered saline (PBS) for 30 min at room temperature. This was followed by rinsing them with PBS, dehydration in 30% sucrose, and embedding in optimal cutting-temperature cryostat embedding medium. Using a cryostat, 10 µm retinal sections were prepared and transferred to Superfrost/plus microscope slides. The slides were stored at −80 °C.

Human donors: Eye globes from human donors with documented diabetic retinopathy, enucleated within 5–8 h of death, were obtained from the Eversight Eye Bank (Ann Arbor, MI, USA). The diabetic retinopathy group included eight donors (3 male and 5 female, 53–78 years of age with 15–32 years of diabetes), and the age-matched nondiabetic control group had seven donors (3 male and 4 female). Tissue exclusion criteria included donors with any other ocular diseases, chronic diseases (e.g., Alzheimer’s disease, cancer, or HIV) and drug use or smoking within the three years prior to their death [31]. The eye globes were coded without any patient identification and were exempted from “informed consent” requirements by the Institutional Review Board Administration.

Retinal microvessels: Retina (1 mouse retina or 1/6th–1/8th of a human retina) were incubated in 5–10 mL deionized water for 60 min at 37 °C in a shaking water bath, and retinal microvessels were isolated under a dissecting microscope [31].

Gene expression: Total RNA was extracted from HRECs/retinal microvessels using TRIzol reagent (Invitrogen, Life Technologies, Carlsbad, CA, USA) according to the manufacturer’s protocol, followed by treatment with RNase-free DNase I (cat. no. M6101; Promega Biotech, Madison, WI, USA). cDNA was synthesized from 1 μg RNA using a high-capacity cDNA reverse transcription kit (cat. no. 4368814; Thermo Fisher Scientific, Waltham, MA, USA), and mRNA levels were determined by quantitative real-time polymerase chain reaction (qRT-PCR) using SYBR green master mix and gene-/species-specific primers (Table 1). *β-actin* (human) or 18S rRNA (mice) was used as the housekeeping gene, and the relative fold change was calculated using the delta–delta Ct method [26,31].

For Lnc*CytB* expression, strand-specific qPCR was performed using the Lnc*CytB* antisense primer with an adapter during cDNA synthesis to avoid the simultaneous quantification of *CytB* mRNA, as described recently [26].

Immunofluorescence: Mitochondrial localization of MRPP1 was performed by the immunofluorescence technique using antibodies against the mitochondrial marker CoxIV (cat. no. AB33985; Abcam Inc., Waltham, MA, USA; 1:200 dilution) and MRPP1 (cat. no. 29087-1-AP; Thermo Fisher Scientific; 1:200 dilution). Secondary antibodies included Alexa Fluor-488 (green) conjugated anti-rabbit (cat. no. A11008; Molecular Probes-Life Technologies, Grand Island, NE, USA; 1:500 dilution) and Texas red-conjugated anti-mouse (cat. no. TI2000; Vector Laboratories, Burlingame, CA, USA; 1:500 dilution). To counterstain the nuclei, immunolabelled cells were mounted using 4′,6-diamidino-2-phenylindole (DAPI)-containing (blue) Vectashield mounting medium (cat. no. H-1000; Vector Laboratories). The images were captured by a Zeiss ApoTome fluorescence microscope (Carl Zeiss, Chicago, IL, USA) using a 63× oil objective and the Apotome module. The fluorescence intensity and Pearson’s correlation coefficient were quantified using Zeiss software module, v. ZEN Pro 2.6.76 [26,31].

RNA immunoprecipitation (RIP): The interaction between MRPP1 and Lnc*CytB* was determined by RIP assay, as described previously [26,31]. Crosslinked samples were sonicated, and agarose gel electrophoresis was performed to confirm uniform shearing. Protein–RNA complexes were immunoprecipitated by 4 μg anti-MRPP1 antibody (cat. no. A304-390A; Thermo Fisher Scientific) or with control anti-IgG (cat. no. ab172730; Abcam). Protein A/G PLUS-Agarose beads (cat. no. sc-2003; Santa Cruz Biotechnology, Santa Cruz, CA, USA) were used to precipitate antibody–chromatin complexes, followed by de-crosslinking of the RNA–protein complexes by proteinase K. RNA was then extracted using TRIzol, cDNA was synthesized and the enrichment of Lnc*CytB* was quantified by strand-specific RT-PCR.

RNA fluorescence in situ hybridization (RNA-FISH): The RNA-FISH immunofluorescence technique was used to co-localize MRPP1 and Lnc*CytB* using anti-MRPP1 antibody- and fluorescein-12-dUTP-incorporated Lnc*CytB* probes. Asymmetric PCR was performed to synthesize the probes, and the QIAquick gel extraction kit (cat. no. 28704; Qiagen, Germantown, MD, USA) was used to purify them. Briefly, HRECs fixed with paraformaldehyde (4% *w*/*v*), dehydrated with 70–100% ethanol and air-dried, were incubated at 37 °C for three hours with the denatured probes in probe hybridization buffer (10% dextran sulphate, 10% formamide and 4× saline–sodium citrate (pH 7.0)). The cells were then washed with the hybridization buffer, followed by PBS. The immunofluorescence technique using anti-MRPP1 antibody followed by Texas red-labeled anti-rabbit secondary antibody (cat. no. TI-1000-1.5; Vector Laboratories; 1:500 dilution) was performed for co-localization. The coverslips were mounted, and the images were captured by a Zeiss ApoTome fluorescence microscope at 63× oil objective using the Apotome module [26,31].

For mouse retina, cryosections were hybridized using the fluorescein-12-dUTP-incorporated Lnc*CytB* probe at 62 °C for six hours [32]. Slides were washed and processed for immunofluorescence staining using anti-MRPP1 antibody (cat. no. 29087-1-AP; Thermo Fisher Scientific; 1:200 dilution) as a primary antibody and Texas red-conjugated secondary antibody (cat. no. TI200; Vector Laboratories; 1:500 dilution). Slides were mounted using DAPI-containing mounting blue medium (Vector Laboratories) and visualized using a Zeiss ApoTome fluorescence microscope using a 20× objective.

Mitochondrial ROS: Reactive oxygen species were quantified by the fluorometric method by incubating 10 µg mitochondrial protein with 5 µM MitoSox red (cat. no. M36008; Invitrogen) for 30 min at 37 °C in the dark. Fluorescence was measured at 500 nm excitation and 580 nm emission wavelengths, and fold changes were calculated considering the values obtained for cells in normal glucose as one [33].

Mitochondrial ROS were also quantified by flow cytometry by staining the cells with 1 μM MitoSox red for 30 min at 37 °C in the dark. After gently washing the cells three times with 37 °C-prewarmed flow buffer (0.5% BSA in PBS), they were scanned at the FL3 640 nm wavelength in a BD Accuri C6 plus flow cytometer (BD Biosciences, San Jose, CA, USA). Raw flow cytometry standard files were analyzed by FlowJo v10.8.1 software (BD Biosciences).

Mitochondrial nucleoids: Mitochondrial nucleoid staining was performed on the coverslips; briefly, cells were incubated with 1× SYBR gold DNA stain (cat. no. S11494; Thermo Fisher Scientific) for 30 min, and after washing the coverslips with PBS, they were fixed with 4% paraformaldehyde for 15 min. This was followed by washing with PBS (4 × 5 min each) and mounting them. The cells were imaged using a 488 nm wavelength filter and a 63× oil objective lens on a Zeiss ApoTome fluorescence microscope [26,34]. The number of nucleoids was counted in 5 to 8 images/group/experiment using the ImageJ software (ImageJ, v. 1.54i, U.S. National Institutes of Health, Bethesda, MD, USA).

Mitochondrial copy numbers: RNA-free genomic DNA was extracted from HRECs using a DNeasy kit (cat. no. 69504; Qiagen, Valencia, CA, USA) as per the manufacturer’s instructions, and mtDNA copy numbers were analyzed by qRT-PCR-based amplification of mitochondrial genome-encoded *CytB* and nuclear genome-encoded *β-actin* using DNA primers (Table 1) [26].

Cell death: The presence of oligonucleosomes in cytosol was determined using monoclonal antibodies directed against DNA and histones and quantifying the histone-associated DNA fragments by employing the Cell Death Detection ELISA PLUS kit (cat. no. 11774425001; Roche Diagnostics, Indianapolis, IN, USA). The results were calculated as fold changes, considering the values obtained for cells in normal glucose as one.

Statistical analysis: Graph Pad Prism v. 10 (San Diego, CA, USA) was used to perform the statistical analysis, and the results are presented as means ± SDs. The significance of variance between multiple groups was calculated using one-way ANOVA, and statistical comparisons between two groups were analyzed by two-tailed Student’s *t*-tests. A *p*-value < 0.05 was considered statistically significant.

## 3. Results

### 3.1. In Vitro Model

**Figure 1:**
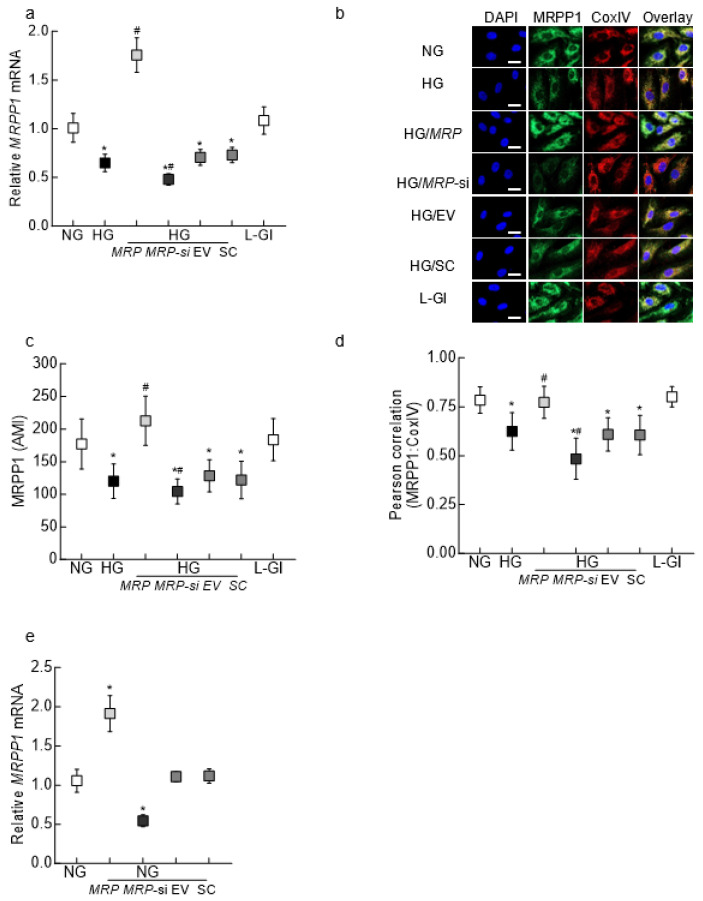
Effect of high glucose on MRPP1 expression in retinal endothelial cells. (**a**) *MRPP1* gene transcripts quantified by qRT-PCR using β-actin as a housekeeping gene. (**b**) Representative images showing MRPP1 mitochondrial localization using Alexa Fluor 488 (green)-conjugated and Texas Red (red)-conjugated secondary antibodies for MRPP1 and CoxIV, respectively. The line marker represents 10 μm. (**c**) AMI of MRPP1, calculated by quantifying the intensity of the green fluorescence. (**d**) Pearson’s correlation coefficient between MRPP1 and CoxIV, calculated using the colocalization software module. (**e**) Relative *MRPP1* mRNA levels, quantified by qRT-PCR. Each measurement was made in duplicate/triplicate in 3–4 different cell preparations, and the values obtained for NG are considered as 1. The values are presented as means ± SDs. NG and HG = cells in 5 mM and 20 mM D-glucose, respectively; HG/*MRP*, HG/*MRP*-si, HG/EV and HG/SC = cells transfected with *MRPP1*-overexpressing plasmids, *MRPP1*-siRNA, an empty vector and control scrambled RNA, respectively, and incubated in 20 mM D-glucose; L-Gl = 20 mM L-glucose. * *p* < 0.05 compared to NG; # *p* < 0.05 compared to HG.

MRPP1 expression and its mitochondrial localization: Compared to HRECs in normal glucose, high glucose downregulated *MRPP1* gene transcripts by ~50% (*p* < 0.05; Figure 1a). In addition to the gene transcripts, MRPPI (green) fluorescence intensity was also significantly lower in cells in high glucose (Figure 1b), and the arithmetic mean intensity (AMI) of MRPP1 was reduced by ~50% (Figure 1c). The Pearson correlation coefficient between MRPP1 and CoxIV was 40% less in cells in high glucose compared to cells in normal glucose, suggesting decreased mitochondrial expression (Figure 1d). *MRPP1* overexpression ameliorated glucose-induced decrease in MRPP1 expression; gene transcripts and fluorescence intensity were significantly higher in *MRPP1*-overexpressing cells in high glucose compared to untransfected cells or cells transfected with an empty vector in high glucose. In contrast, *MRPP1*-siRNA-transfected cells in high glucose exhibited a significant reduction in MRPP1 gene transcripts, AMI and the MRPP1-CoxIV Pearson correlation coefficient compared to untransfected cells in high glucose (*p* < 0.05). As an osmotic control, untransfected cells incubated in 20 mM L-glucose showed similar MRPP1 expression and mitochondrial localization to cells in normal glucose (Figure 1a–d). Figure 1e shows ~2.5 times more *MRRP1* mRNA in HRECs transfected with *MRPP1*-overexpressing plasmids and 50% less in cells transfected with *MRPP1*-siRNA compared to untransfected cells or cells transfected with an empty vector or scrambled control RNA.

**Figure 2:**
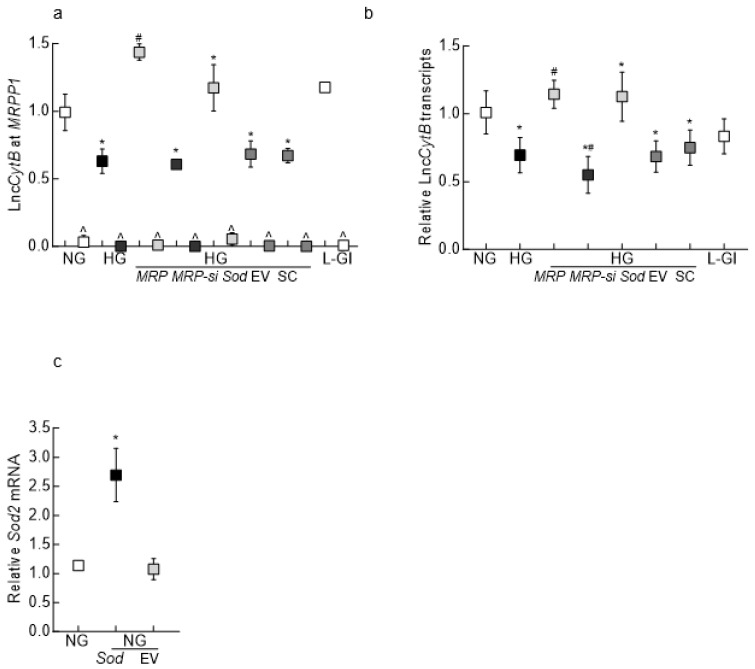
Effect of high glucose on MRPP1–Lnc*CytB* interaction. (**a**) Lnc*CytB*–MRPP1 interaction, quantified in HRECs by the RIP technique using IgG (^) as an antibody control. (**b**) Lnc*CytB* transcripts analyzed by strand-specific PCR using β-actin as a housekeeping gene. (**c**) Relative gene transcripts of *Sod2*, measured by qRT-PCR. Each measurement was made in 3–4 different cell preparations, and the values are presented as means ± SDs. NG = 5 mM D-glucose; HG = 20 mM D-glucose; HG/*MRP*, HG/*MRP-si*, HG/*Sod-*, HG/EV and HG/SC = HRECs transfected with *MRPP1*-overexpressing plasmids, *MRPP1*-siRNA, *Sod2* overexpressing plasmids, an empty vector and scrambled control RNA, respectively, and incubated in high glucose; L-Gl = 20 mM L-glucose. * *p* < 0.05 compared to NG; # *p* < 0.05 compared to HG.

Interaction between MRPP1 and Lnc*CytB*: To confirm the interaction between MRPP1 and Lnc*CytB*, RIP was performed. Compared to normal glucose, high glucose decreased MRPP1–Lnc*CytB* interaction, and in the same experiment, the control’s IgG binding was <1% (indicated with ^; Figure 2a). Furthermore, as expected [26,31], the gene transcripts of Lnc*CytB* were also significantly decreased in high glucose compared to normal glucose (Figure 2b). While *MRPP1* overexpression ameliorated glucose-induced decrease in MRPP1–Lnc*CytB* interaction and downregulation of Lnc*CytB* transcripts, *MRPP1*-siRNA further decreased Lnc*CytB* transcripts, and the values for si-RNA-transfected cells were significantly lower compared to untransfected cells in high glucose.

Since Lnc*CytB* is intimately associated with mitochondrial stability [26,35], the effect of overexpression of *Sod2* on MRPP1–Lnc*CytB* interaction was determined. *Sod2* overexpression, in addition to preventing a decrease in Lnc*CytB* expression, ameliorated glucose-induced decrease in MRPP1–Lnc*CytB* interaction, and the values obtained for *Sod2*-overexpressing cells in high glucose were significantly different from those obtained for untransfected cells in high glucose (Figure 2a,b). Figure 2c is included to show an ~2-fold increase in *Sod2* mRNA in cells transfected with *Sod2*-overexpressing plasmids compared to cells with the empty vector.

**Figure 3:**
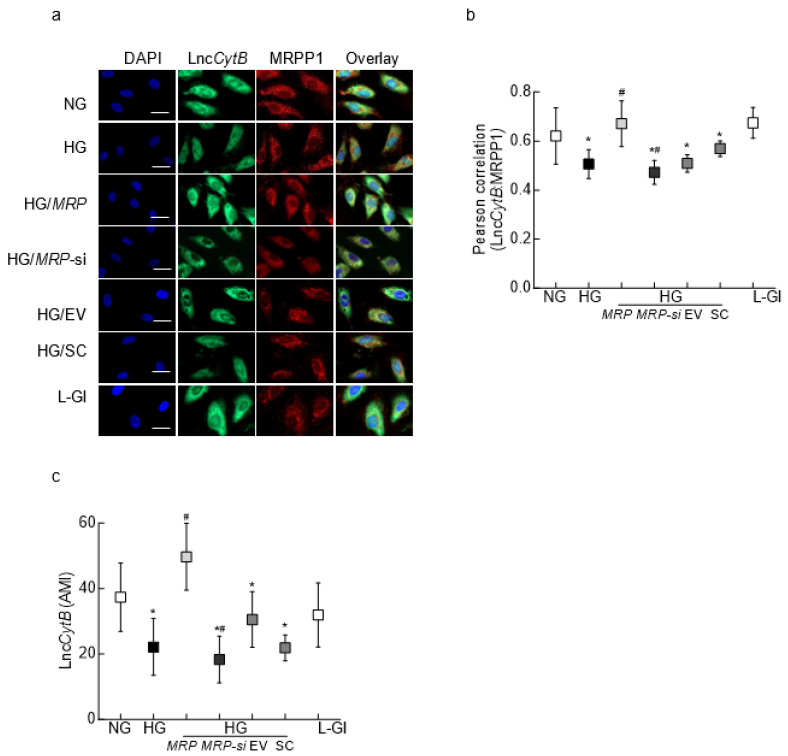
MRPP1–Lnc*CytB* interaction determined by RNA-FISH. (**a**) Representative RNA-FISH images with fluorescein 12-dUTP-labeled Lnc*CytB* probe in green and Texas Red-conjugated secondary antibody against MRPP1 in red (size marker = 10 μm). (**b**) Pearson correlation coefficient between Lnc*CytB* and MRPP1. (**c**) Arithmetic mean fluorescence intensity (AMI) of Lnc*CytB*. Each measurement was made in 5–7 cells and repeated in 2–3 different cell preparations. The values in the graphs are presented as means ± SDs. NG and HG = cells in 5 mM and 20 mM D-glucose, respectively; HG/*MRP*, HG/*MRP-si*, HG/EV and HG/SC = cells transfected with *MRPP1*-overexpressing plasmids, *MRPP1*-siRNA, an empty vector and scrambled RNA, respectively, and incubated in 20 mM D-glucose; L-Gl = 20 mM L-glucose. * *p* < 0.05 compared to NG; # *p* < 0.05 compared to HG.

Glucose-induced alteration of MRPP1–Lnc*CytB* interaction was further confirmed by the RNA-FISH technique; compared to normal glucose, co-staining of MRPP1 and Lnc*CytB* was significantly decreased, and Pearson’s correlation coefficient of MRPP1 and Lnc*CytB* was reduced by ~50% in high glucose (Figure 3a,b). While *MRPP1* overexpression ameliorated the glucose-induced decrease in co-staining of Lnc*CytB* and MRPP1, *MRPP1*-siRNA further reduced it, as demonstrated by the MRPP1 and Lnc*CytB* Pearson’s correlation coefficient. Consistent with the transcripts, glucose-induced decrease in Lnc*CytB* fluorescence intensity (green) and the AMI of the green fluorescence were also prevented by *MRPP1*-overexpressing plasmids but were further worsened by *MRPP1*-siRNA (Figure 3a,c). However, transfection with an empty vector or scrambled RNA had no effect on glucose-induced decrease in Lnc*CytB*–MRPP1 interaction or Lnc*CytB* fluorescence, and the values were not different from those obtained for untransfected cells in high glucose (*p* > 0.05). Untransfected cells incubated with 20 mM L-glucose or in 5 mM D-glucose had similar values (Figure 3).

**Figure 4:**
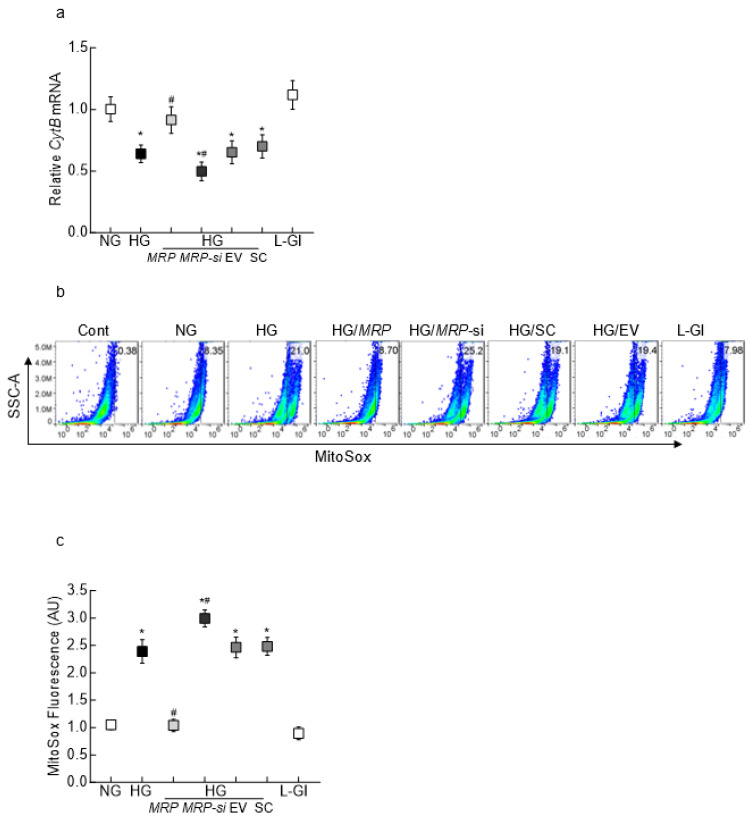
*MRPP1* overexpression and high-glucose-induced mitochondrial dysfunction in retinal endothelial cells. (**a**) *CytB* gene transcripts quantified by RT-PCR using β-actin as a housekeeping gene. Mitochondrial ROS measured by (**b**) flow cytometry using MitoSox and (**c**) by quantifying MitoSox fluorescence intensity using isolated mitochondria, considering values obtained for the NG group as 1. The values are presented as means ± SDs, obtained from 3–4 different cell preparations, with each measurement made in duplicate. * *p* < 0.05 vs. NG; # *p* < 0.05 vs. HG.

MRPP1–Lnc*CytB* interaction and mitochondrial damage: Since downregulation of Lnc*CytB* decreases cytochrome B complex III activity and increases ROS [35], the effect of *MRPP1* overexpression on glucose-induced downregulation of gene transcripts of mtDNA-encoded cytochrome B (*CytB*, an integral component of complex III) and mitochondrial ROS levels was investigated. *MRPP1* overexpression ameliorated glucose-induced downregulation of *CytB* transcription, and *MRPP1*-siRNA further decreased it, further suggesting its role in mtDNA stability (Figure 4a). The values for high-glucose-exposed *MRPP1*-overexpressing cells, *MRPP1*-siRNA-transfected cells and untransfected cells were significantly different from each other (*p* < 0.05). Similarly, mitochondrial ROS were also decreased in high-glucose-exposed *MRPP1*-overexpressing cells but were further increased in *MRPP1*-siRNA-transfected cells (Figure 4b,c).

**Figure 5:**
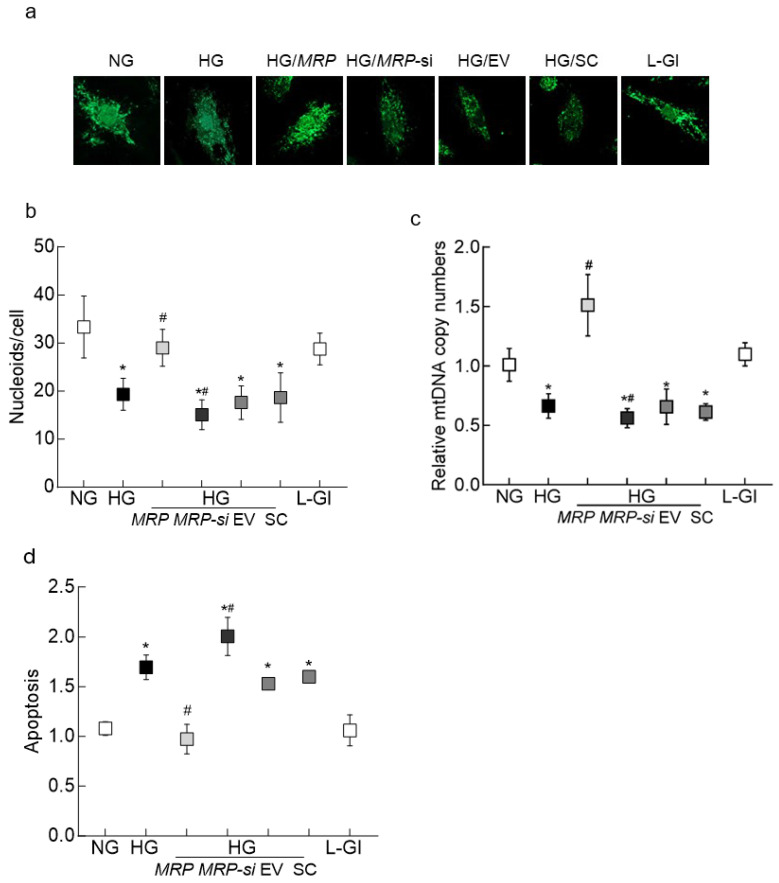
*MRPP1* regulation and high-glucose-induced mtDNA damage and cell apoptosis. (**a**) Representative images showing SYBR gold staining for nucleoids and (**b**) the number of nucleoids from 5–8 images/group/experiment, quantified using ImageJ software. (**c**) mtDNA copy numbers, quantified in genomic DNA, and (**d**) cell apoptosis. Values presented as means ± SDs, with each measurement made in duplicate in 3–4 different cell preparations. NG = 5 mM D-glucose; HG = 20 mM D-glucose; HG/*MRP*, HG/*MRP-si*, HG/EV and HG/SC = cells transfected with *MRPP1*-overexpressing plasmids, *MRPP1*-siRNA, an empty vector and scrambled RNA, respectively, and incubated in 20 mM D-glucose; L-Gl = 20 mM L-glucose. * *p* < 0.05 compared to NG; # *p* < 0.05 compared to HG.

Consistent with mitochondrial ROS and *CytB* expression, while *MRPP1* overexpression attenuated glucose-induced decrease in the protective mtDNA nucleoids, *MRPP1*-siRNA further decreased them (Figure 5a,b). The average number of nucleoids per cell was ~32 in normal glucose, which decreased to ~18 in high glucose; however, *MRPP1*-overexpressing cells incubated in high glucose had ~30 nucleoids/cell and *MRPP1*-siRNA-transfected cells had ~15, further confirming the effect of MRPP1-Lnc*CytB* on mitochondrial stability. Furthermore, while *MRPP1* overexpression ameliorated glucose-induced decrease in mtDNA copy numbers, its siRNA further decreased them (Figure 5c). Similarly, glucose-induced increase in cell apoptosis was attenuated by *MRPP1* overexpression and was exacerbated by its siRNA (Figure 5d). The values obtained for *MRPP1*-overexpressing cells and siRNA-transfected cells in high glucose were significantly different from each other (*p* < 0.05); untransfected cells in 5 mM D-glucose and in 20 mM L-glucose, however, showed similar values (*p* > 0.05).

### 3.2. Mouse Retinal Microvessels

**Figure 6:**
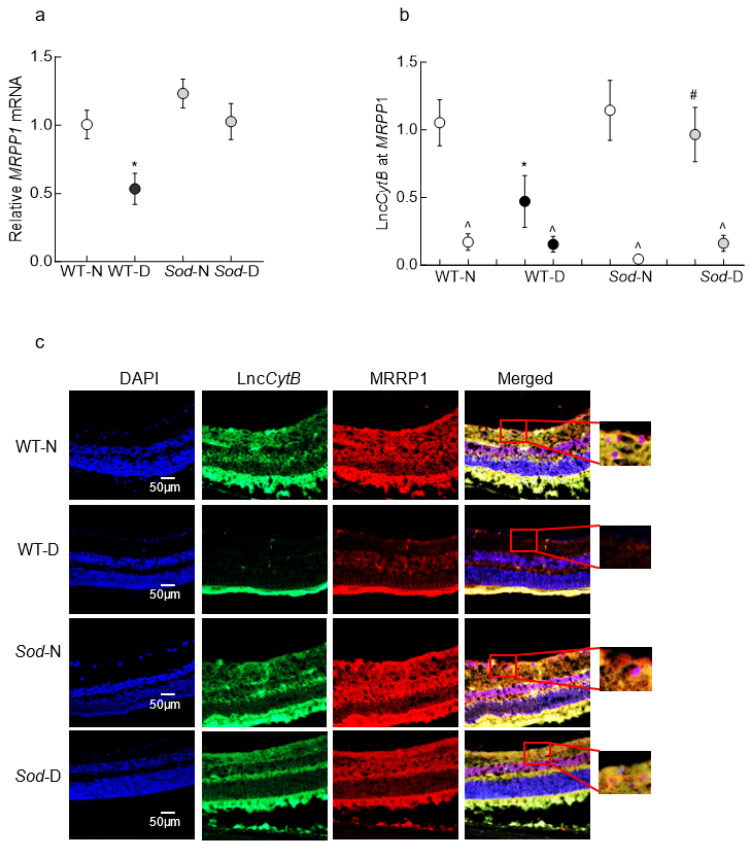
Effect of diabetes on MRPP1–Lnc*CytB* interactions in mouse retinal microvessels. Microvasculature from mouse retina was analyzed for (**a**) *MRPP1* gene transcripts by qRT-PCR using 18S rRNA as a housekeeping gene and (**b**) Lnc*CytB* at MRPP1 by the RIP technique using IgG (^) as an antibody control. WT-N group values are considered as 1. (**c**) Representative images showing co-localization of Lnc*CytB* and MRPP1 in retinal cryosection using fluorescein 12-dUTP-labeled Lnc*CytB* probe (green) and Texas Red (red)-conjugated secondary antibody against MRPP1. Values in graphs are presented as means ± SDs obtained from 5–8 mice in each of the four experimental groups. WT-N and WT-D = wildtype normal and diabetic mice; *Sod*-N and *Sod*-D = *Sod*2-overexpressing normal and diabetic mice; * *p* < 0.05 compared to WT-N, # *p* < 0.05 compared to WT-D mice.

In accordance with our in vitro results, the retinal microvasculature from wildtype diabetic mice (WT-D) exhibited a 40% decrease in *MRPP1* gene transcripts compared to age-matched normal mice (WT-N). In the same diabetic mice, the binding occupancy of MRPP1-Lnc*CytB* also decreased by >50%. However, in *Sod2*-overexpressing diabetic mice (*Sod*-D), the mice that were protected from downregulation of Lnc*CytB* and the development of diabetic retinopathy [26,35], relative *MRPP1* gene transcripts were similar to those in the WT-N group. Similarly, although MRRP1 and Lnc*CytB* interaction was decreased in the WT-D group, *Sod2* overexpression prevented this diabetes-induced decrease, and the values for the WT-N, *Sod*-N and *Sod*-D groups were not significantly different from each other (*p* > 0.05). Compared to the values obtained for MRRP1 antibodies, normal rabbit IgG values for all of the samples were <1% (Figure 6a,b). Consistent with the RIP assay, RNA in situ hybridization also presented decreased co-expression of MRPP1 and Lnc*CytB* in diabetic mice (the WT-D group), as shown by decreased co-staining of MRPP1 and Lnc*CytB*. However, mice in the *Sod*-D group showed significantly higher co-staining of MRPP1 and Lnc*CytB* compared to mice in the WT-D group (Figure 6c).

### 3.3. Human Retinal Microvessels

**Figure 7:**
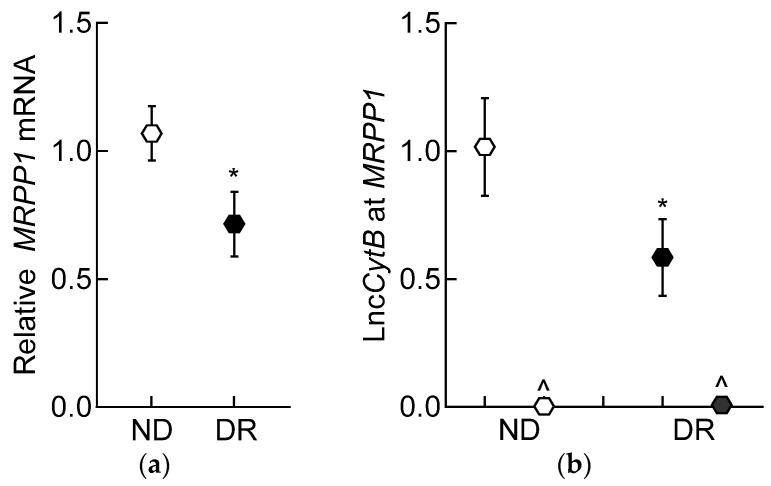
MRPP1–Lnc*CytB* interaction in human donors with diabetic retinopathy. Retinal microvessels were analyzed (**a**) for *MRPP1* mRNA by qRT-PCR using *β-actin* as a housekeeping gene and (**b**) Lnc*CytB*–MRPP1 interactions by the RIP technique using IgG (^) as an antibody control. Values for the ND group are considered as 1 and are presented as means ± SDs of six or more donors in each group, with each measurement made in triplicate. DR = documented diabetic retinopathy donors; ND = age-matched nondiabetic donors (ND); * *p* < 0.05 vs. ND.

MRPP1 and its interaction with Lnc*CytB* were also investigated in the retinal microvessels from human donors with established diabetic retinopathy (the DR group). In addition to reduced levels of Lnc*CytB* transcripts in human donors with diabetic retinopathy compared to the age-matched nondiabetic donors [26], gene transcripts of *MRPP1* and its interaction with Lnc*CytB* were also significantly decreased in donors with diabetic retinopathy (Figure 7).

## 4. Discussion

Mitochondrial dysfunction is intimately associated with the development of diabetic retinopathy, and the damage to mtDNA initiates a self-propagating vicious cycle of free radicals [3,5]. The double-stranded circular mtDNA, which consists of heavy and light strands, transcribes polycistronic precursors of mitochondrial transcripts covering almost the entire length of both strands [14,36,37]. Coding genes in the long precursor transcripts are interspersed with one or more tRNAs acting as “punctuation” marks, and MRPPs are responsible for processing the 5′ ends while mitochondrial RNase Z is responsible for processing the 3′ ends of tRNAs [24,38]. Although MRPPs catalyze the cleavage of mitochondrial precursor tRNAs, in contrast to their nuclear counterparts, MRPPs do not have any RNA component [27,30,39]. The MRPP complex has three subunits: a subcomplex formed between MRPP1 and MRPP2 directs the MRPP3 nuclease domain to the cleavage site, which increases the rate and accuracy of cleavage [40,41]. MRPP1 has also been shown to play an important role in the accumulation of mtDNA-encoded lncRNAs and in the maturation and abundance of Lnc*CytB* and Lnc*ND5* [24,27]. Our recent work has demonstrated a crucial role of Lnc*CytB* in diabetic retinopathy and implicated its downregulation in hyperglycemic milieus in mitochondrial functional and genomic instability [26,35]. Here, our results show that MRPP1 regulates *LncCytB* expression in hyperglycemia; high glucose decreases MRPP1 expression and its mitochondrial accumulation in retinal endothelial cells and also reduces its interactions with Lnc*CytB*. While overexpression of *MRPP1* prevents an increase in mitochondrial ROS and capillary cell apoptosis and stabilizes mtDNA by inhibiting decreases in mtDNA copy numbers and protective nucleoids, *MRPP1*-siRNA further aggravates mitochondrial damage. Consistent with our in vitro results, the retinal vasculature of diabetic mice showed a significant reduction in Lnc*CytB* and MRPP1 interaction. Overexpression of *Sod2*, which protects the retina from increased accumulation of mitochondrial superoxide and the development of diabetic retinopathy [13], also prevents reductions in MRPP1 and its interactions with Lnc*CytB* and restores Lnc*CytB* expression. Similar decreases in MRPP1 expression and its interaction with Lnc*CytB* in human donors with documented diabetic retinopathy further confirm the important role of MRPP1 in the regulation of Lnc*CytB* expression.

As stated above, mitochondrial stability requires over 1500 proteins, but the mitochondrial genome encodes for only 13 polypeptides [14,42], making the nuclear genome an integral component of mitochondrial stability. Nuclear DNA-encoded MRPPs are considered necessary for the accumulation of mtDNA-encoded lncRNAs, and Lnc*CytB* is a mitochondrial genome-encoded lncRNA. Although these MRPPs lack RNA components essential for catalytic function, they cleave the 5′ ends of tRNAs and initiate the maturation of the precursors [27,30], and defects in MRPPs, in addition to aberrant mitochondrial tRNA processing, have been shown to cause mitochondrial dysfunction [43]. Among the three subunits, MRPP1 is a methyltransferase, MRPP2 is a multifunctional protein associated with amino acid catabolism and lipid metabolism, and MRPP3 cleaves mitochondrial tRNAs at the 5′ end [38,40]. Although knockdown of *MRPP3* alone decreases lncRNAs, the effect is relatively less dramatic compared with *MRPP1* knockdown. In HeLa human cervical cancer cells, MRPP1 is considered necessary for the maturation and abundance of mtDNA-encoded lncRNAs [24,27]. Here, our results show that MRPP1 levels are decreased in the mitochondria in hyperglycemic milieus, and overexpression of *MRPP1*, not the empty vector, ameliorates glucose-induced decreases in MRPP1–Lnc*CytB* interactions and Lnc*CytB* expression, but *MRPP1*-siRNA further worsens them. In support of this, downregulation of Lnc*CytB* in diabetic retinopathy is considered to contribute to mtDNA vulnerability to damage and also impairs oxygen consumption rate [24,27].

Dysfunctional/damaged mitochondria lead to apoptosis of retinal capillary cells—a phenomenon that precedes the development of retinopathy [5]—and Lnc*CytB* is intimately associated with mitochondrial functional stability [35]. Overexpression of Lnc*CytB* has been shown to attenuate mitochondrial ROS production, and it also protects mitochondrial genome stability by preventing decreases in protective nucleoids [26,35]. The results presented here clearly show that *MRPP1* overexpression can duplicate the effects of Lnc*CytB* overexpression on mitochondrial functional and genomic stability by preventing increases in mitochondrial ROS and decreases in mtDNA copy numbers and protective nucleoids. In addition, *MRPP1* overexpression also ameliorates mtDNA transcription, which is downregulated in high-glucose conditions, as shown by *CytB* gene transcripts. The role of MRPP1 in the regulation of Lnc*CytB* mitochondrial stability is further strengthened by our results showing attenuation of capillary cell apoptosis in *MRPP1*-overexpressing HRECs.

In accordance with retinal endothelial cells in high-glucose conditions, results from in vivo models using retinal microvessels from diabetic mice also show reduced levels of MRPP1 and MRPP1–Lnc*CytB* interaction; in support of this, in diabetic retinopathy, Lnc*CytB* expression is decreased in retinal microvessels [26]. Furthermore, we also showed that overexpression of *Sod2* protects against diabetes-induced decreases in MRPP1 and its interactions with Lnc*CytB*; *Sod2*-overexpressing diabetic mice are also protected from decreases in retinal Lnc*CytB* and mtDNA nucleoids and increases in ROS and do not develop retinopathy [13,26], supporting the role of MRPP1–Lnc*CytB* in diabetic retinopathy.

The results from the experimental models were further confirmed by similar decreases in MRPP1 expression and its interactions with Lnc*CytB* in the retinal microvasculature of human donors with documented diabetic retinopathy, further strengthening the role of MRPP1–Lnc*CytB* in mitochondrial homeostasis diabetic retinopathy.

Mitochondrial RNAs are produced by bidirectional transcription of the circular mtDNA in long polycistronic precursor transcripts, and we acknowledge that mitochondria are also rich in double-stranded RNAs [44,45]. Our study focused on the role of MRPP1 in Lnc*CytB* regulation mitochondrial homeostasis, but the possibility that damaged mitochondria could leak double-stranded RNAs into the cytosol, leading to innate immunity or the activation of apoptosis [46,47], cannot be ruled out.

**Figure 8:**
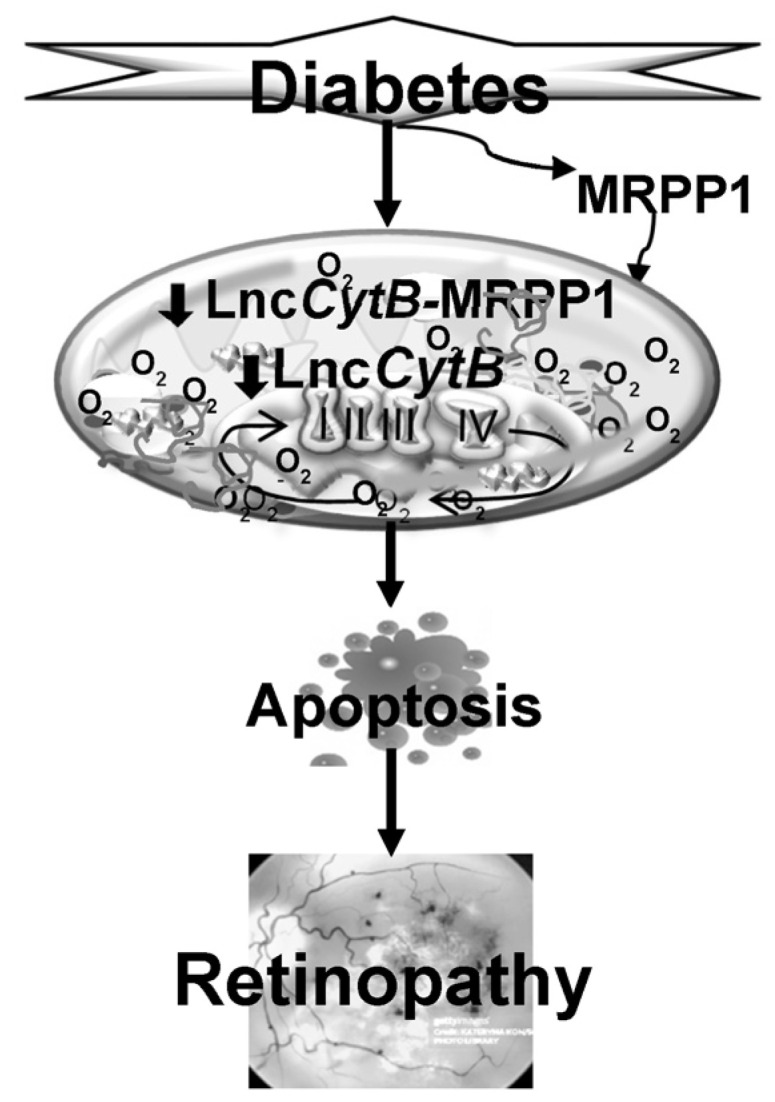
Downregulation of MRPP1 in diabetes leads to decreased processing of mitochondrial RNA, resulting in the downregulation of LncCytB. Reduced levels of LncCytB results in reduction in mitochondrial nucleoids, leading to decreased mitochondrial genomic stability and impaired electron transport chain system, which ultimately culminates in the development of diabetic retinopathy.

In summary, MRPP1 plays an important role in the processing and the regulation of expression of Lnc*CytB*—a mitochondrial genome-encoded lncRNA. Downregulation of MRPP1 in diabetes reduces transcript processing, which results in the downregulation of Lnc*CytB* expression. This culminates in mitochondrial functional and genomic instability, increasing capillary cell death, and ultimately in the development of retinopathy (Figure 8). Thus, preventing MRPP1 downregulation in diabetes will help protect mitochondria and could possibly inhibit the development of retinopathy—a blinding disease which diabetic patients fear the most.

## Figures and Tables

**Table 1:** Primer sequences.

Human RNA	
ssLnc*CytB* antisense	5′-GGTATCTGGACTTCGAAGGCACGAATGACCAACAGGAGGCTAA-3′
Lnc*CytB*	FWD 5′-5′-CCATAGACCTGAAGCTTCCGT-3′
	REV 5′-TATTATAAAGCGGGTGATTCG-3′
*MRPP1*	FWD 5′-GGCAGAGAAGTACCAGAACACATC-3′
	REV 5′-CCCTTGCTGCTTTCATTTC-3′
*CytB*	FWD 5′-ATGGTAGATGTGGCGGGTTT-3′
	REV 5′-TCTCCGATCCGTCCCTAACA-3′
*Sod2*	FWD5′-CCTGCTCCCCGCGCTTTCTT-3′
	REV 5′-CGGGGAGGCTGTGCTTCTGC-3′
*β-actin*	FWD 5′-AGCCTCGCCTTTGCCGATCCG-3′
	REV 5′-TCTCTTGCTCTGGGCCTCGTCG-3′
Human DNA	
*CytB*	FWD 5′-TCACCAGACGCCTCAACCGC-3′
	REV 5′-GCCTCGCCCGATGTGTAGGA-3′
*β-actin*	FWD 5′-CTTTCCTGCCTGAGCTGACC-3′
	REV 5′-CCTAGAAGCATTTGCGGTGG-3′
Mouse	
ssLnc*CytB* antisense	5′-GGTATCTGGACTTCGAAGGCAACCCACAAGATGACCAACCG-3′
Lnc*CytB*	FWD 5′-CCATAGACCTGAAGCTTCCGT-3′
	REV 5′-AATCACACAAATTTTGTACTG-3′
*MRPP1*	FWD 5′-ACCCGCCCCATCCAACATCTCAT-3′
	REV 5′-ACTTGAGAGCCTCTTCCCAGTTA-3′
18S rRNA	FWD 5′-GCCCTGTAATTGGAATGAGTCCACTT-3′
	REV 5′-CTCCCCAAGATCCAACTACGAGCTTT-3′

## Data Availability

RAK is the guarantor of this work and, as such, has full access to all data used in the study and takes responsibility for the integrity of the data and the accuracy of the data analysis.
